# Training social care staff in promoting self-determination and nutritional health for people with intellectual disabilities using 360^o^ virtual reality videos and ethical reflection: a qualitative study

**DOI:** 10.1186/s12913-026-14308-5

**Published:** 2026-03-16

**Authors:** Hege Mari Johnsen, Marianne Hovet Steig, Ellen Margrete Iveland Ersfjord, Mugula Chris Safari, Anne Marit Fone, Kristian Leonard Melby

**Affiliations:** 1 Department of Health and Nursing Science, Faculty of Health and Sport Sciences, University of Agder, PO Box 509, Grimstad, 4898 Norway; 2 Department of Psychosocial Health, Faculty of Health and Sport Sciences, University of Agder, Grimstad, Norway; 3 Unit for Habilitation, Grimstad Municipality, Grimstad, Norway

**Keywords:** Competence, Decision-making, Food, Immersive technology, Nutrition, Ppeople with intellectual disabilities, Self-determination, Staff perspectives, Virtual reality

## Abstract

**Background:**

People with intellectual disabilities living in supported housing facilities are vulnerable to developing obesity and malnutrition. It is therefore essential to strengthen staff competence in providing nutrition and dietary support for people with intellectual disabilities, with particular attention to balancing the provision of appropriate health care with the promotion of residents’ autonomy and self-determination. The aim of this study was to explore social care service providers’ experiences with a teaching and learning approach that combines the use of 360° virtual reality videos with structured ethical reflection focusing on self-determination and preventive dietary work for people with intellectual disabilities.

**Methods:**

This study employed an explorative qualitative design that tested a learning intervention consisting of watching a 360^o^ video followed by participating in a structured group reflection. Data were collected through three focus group interviews with a total of 14 participants from two Norwegian municipality-based supported housing facilities for people with intellectual disabilities. The data were analysed using inductive reflexive thematic analysis.

**Results:**

Three main themes with associated subthemes were identified: (a) the opportunity to experience and immerse oneself in a challenging situation, (b) the opportunity to expand perspectives together, and (c) Satisfaction with the teaching and learning approach. The 360^o^ videos demonstrated immersive qualities, such as authenticity, presence and emotional engagement. Our results also showed that the teaching and learning approach heightened self-awareness, ethical sensitivity, reflection and ethical judgement related to the facilitation of self-determination for people with intellectual disabilities.

**Conclusions:**

Our results showed that the teaching and learning approach of combining 360° videos with structured ethical reflection provided an immersive experience and stimulated meaningful reflections, on which the experiential learning process depends. The participants in our study revealed rich perspectives and strong competence related to supporting residents, showing that staff members can be a potential source for increasing ethical awareness and preventing unethical practices in the support of people with intellectual disabilities regarding food and eating.

**Supplementary Information:**

The online version contains supplementary material available at 10.1186/s12913-026-14308-5.

## Introduction

In Norway, deinstitutionalisation for people with intellectual disabilities has been the key strategy for promoting social inclusion, leading to the replacement of large institutions with supported housing facilities [1, 2]. These facilities typically consist of individual apartments with shared common areas or co-located housing arrangements [3]. Unlike former long-stay institutions, residents of supported housing generally prepare most of their own meals in their apartments independently or with assistance, and may receive support with shopping for groceries [3, 4]. Intellectual disabilities involve cognitive limitations such as difficulties with memory, abstract reasoning, and understanding the possible consequences of one’s choices or behaviour [3, 5], which may make it difficult for individuals to make good lifestyle choices [5]. Thus, despite the good intentions of deinstitutionalising people with intellectual disabilities, individuals in supported housing are especially vulnerable to overweight and malnutrition [3, 5, 6]. Contributing factors include unhealthy diets, sedentary lifestyles, genetic predispositions (e.g., Prader-Willi and Down syndrome), and side effects of antipsychotic medications [7]. Obesity increases the risk of noncommunicable diseases, including some cancers [8], diabetes and cardiovascular diseases [9], highlighting the need for preventive efforts to reduce avoidable morbidity and premature mortality in this population.

Several studies have shown that staff in supported housing facilities caring for people with intellectual disabilities often lack knowledge of and routines related to food and nutrition [3–5, 10]. A Norwegian report on staff competence in services for people with intellectual disabilities found that less than a third (28%) of staff had a university or college education, and approximately one third lacked any formal qualifications [11]. The same report indicated that a lack of competence among staff affects both service quality and residents’ rights. Other factors influencing staff’s ability to promote a healthy diet among adults with intellectual disabilities include staff attitudes, a lack of shared routines [3, 5] and staff interpretations of self-determination and autonomy [5, 10]. Such interpretations may conflict with staff’s duty to promote a healthy lifestyle and prevent harm to residents’ health [12]. These challenges are further complicated by the legal framework governing coercion and consent. While the Norwegian Health and Care Services Act, Chap. 9, regulates coercive measures related to behaviour, it does not cover medical treatment provided against a person’s will [12]. In cases where individuals lack decision-making capacity and oppose medical treatment that may prevent significant health damage, such interventions are instead regulated by the Norwegian Patient Rights Act [12]. Although the law distinguishes clearly between medical treatment and behaviour modification, this distinction may be less clear in practice, particularly in dietary and lifestyle-related interventions [12]. As a result, staff may face uncertainty when health-related concerns are closely intertwined with behavioural issues, potentially limiting effective responses [12]. Consistent with this, a recent Norwegian study found that limiting food access through coercion failed to address the underlying causes of obesity and malnutrition among people with intellectual disabilities [12].

Promoting the self-determination of people with intellectual disabilities is now regarded as best practice [13], emphasizing their role as the primary causal agents in their own lives and their right to make decisions affecting their quality of life without undue external influence or interference [13]. This perspective aligns with international and national regulations protecting and promoting the rights of people with disabilities [14–17] and with contemporary relational understandings of intellectual disabilities, which emphasize interactions between individuals and their social and cultural contexts [16]. Self-determination should not, however, be interpreted to mean that individuals with intellectual disabilities must always make decisions on their own, without support from staff or other caregivers. For example, if a person is situated within an obesogenic environment and experiences challenges related to weight and weight-related comorbidities, there is also a scope for employing ‘informal coercion’ by staff, a form of mild coercion not legally defined as coercion [18, 19] to promote a health promoting diet. Informal coercion involves persisting with an initiative or action despite acting against the service-users’ expressed wishes, without crossing into formal coercion [19]. To apply such influence appropriately, staff require knowledge of diet and nutrition in people with intellectual disabilities and a good understanding of the service-users’ right to self-determination in food related decisions [5, 6]. Staff must also be able to reflect on ethical dilemmas and make health-promoting decisions in accordance with relevant legislation [19, 20].

The use of immersive environments, such as VR headsets with 3D or 360° video content, in health and social care education has demonstrated potential to enhance learning outcomes, motivation and interest in learning [21–25], foster empathy [25–27] and develop ethical reflection skills [28]. Although the use of VR (including 360° videos) is increasing within social care education [22, 27–31] and various healthcare settings [32], research remains limited to their application within social care services to support staff development of skills such as professional reflection and practice in the care of people with intellectual disabilities. This study addresses this gap by exploring social care service providers’ experiences with a learning intervention combining 360° videos and structured ethical reflection, focusing on self-determination and preventive dietary practices for people with intellectual disabilities. Accordingly, the research question is: How do social care staff experience the use of 360° videos combined with ethical reflection as a tool to enhance the promotion of self-determination and preventive dietary practices for people with intellectual disabilities?

## Experiences and reflection as the source of learning

Our learning intervention (described under the context of this study and the learning intervention) combines the experience of watching 360° videos with structured group-based ethical reflection and is grounded in a combination of experiential and social constructivist learning theory [23, 33]. Experiential learning is defined as ”the process whereby knowledge is created through transformation of experience” [34]. An important component in this process is reflection in- and on action [35]. By watching the 360° video scenarios, our participants are observers of challenging situations in the interaction between a staff member and a resident with intellectual disabilities related to nutrition and self-determination. Reflection in action occurs when participants bring their own knowledge, values, and perspectives to the challenging situations they observe. Watching the 360° videos is followed by a group-based ethical reflection, where the participants are provided with the opportunity to share and discuss their reflections on the situations (reflection on action). Through the different steps of the ethical reflection, the participants need to relate theory to their experiences, reflections and suggested actions, and may internalize acquired knowledge and skills through social construction. Finally, participants may transfer what they have learned through the learning intervention to real clinical practice situations. By combining the experience of watching 360° videos with structured ethical reflection, we view the 360° videos as the immersive component and the structured group reflections as the interactive (and collaborative) component in which the participants explore and test (in collaboration) different lines of ethical reasoning and choices of action [36].

### The context of this study and the learning intervention

In the current study, we explored social care staffs’ experiences with a teaching and learning approach that included the two main components: (1) watching a 360° virtual reality (VR) video and (2) participating in a structured group-based ethical reflection. The context of this study and the two components of the learning intervention will be described below.

This study was conducted in collaboration between a municipality in the south of Norway and the University of Agder. The content of the 360° video scenarios focuses on challenges in the interaction between a staff member and a resident with intellectual disabilities related to nutrition and self-determination. The videos were developed based on insights from three workshops with people with intellectual disabilities living in supported housing facilities, social care service staff and informal carers. The content in the 360° videos therefore reflects perceptions from the involved stakeholders and are embedded in a local- and current historical context. People with intellectual disabilities and social care service providers also contributed as actors in the 360° videos. The people with intellectual disability involved had mild or moderate intellectual disability [37]. The development process resulted in three 360° videos which are described in Table 1 below. The videos show examples of user-led practice, restricted opportunities for choice-making and the expression of preferences and/or informal coercion [13, 19]. The process of developing the 360° videos will be reported in another paper.

**Table 1:** Content of the 360° Video Scenarios

Title of the Scenario (Duration)	Description
1. Lunch (3.26 min)	A staff member helps a resident write a shopping list. They discuss what should be on the shopping list. The resident wants hot dogs for dinner, while the staff member wants him to choose an alternative (chicken).
2. Shopping List (2.35 min)	A staff member enters an apartment to help a resident prepare lunch. She prepares the lunch, which includes cold cuts, vegetables and a limited amount of mayonnaise. She then leaves the apartment. While she is out, the resident finds chocolate spread and finishes his meal. The staff member re-enters the apartment and is distressed.
3. At the Grocery Store (2.53 min)	A resident is at the grocery store to shop with a staff member. They walk through the store to buy what is on the shopping list. There are also other customers in the store. The resident wants to buy potato chips, which are not on the shopping list. A discussion ensues between the resident and the staff member.

After watching the 360^o^ video, the participants were accompanied by a facilitator to another room for the second main component of our learning intervention, the ethical reflection. For this session, we applied the six-step structured model for ethical reflection (see Table 2) developed by the Centre for Medical Ethics (CME) at the University of Oslo [36]. The participants had received an electronic copy of the six-step CME model for systematic reflection in advance and were asked to read it before attending the learning intervention. In addition, each group member was given a hard copy of the reflection model, including supporting texts, before the group reflection started. This helped structure the discussion and made it easier for the groups to follow the steps of the model during the reflection. The CME model served as an analytical tool to support participants in identifying and reflecting on the ethical challenges that were depicted in the VR scenarios.

**Table 2:** The CME structured ethical reflection model

1. What is the ethical problem?
2. What are the facts of the case?
3. Who is involved, and what are their views?
4. Which values, laws and guidelines are relevant?
5. What are the alternative courses of action?
6. Overall assessment.

The learning intervention was carried out at the university campus in a VR laboratory. Some of the authors, who also contributed to the main project, facilitated the learning intervention. The facilitators had tested the equipment and the facilities in advance. On the day of the learning intervention, the participants met at the university campus in the extended reality laboratory. First, they received a joint introduction that lasted approximately 10 min, in which the day’s schedule, practical information about the study, the learning objectives and the structure of the ethical reflection (CME) were presented briefly.

**Fig. 1:**
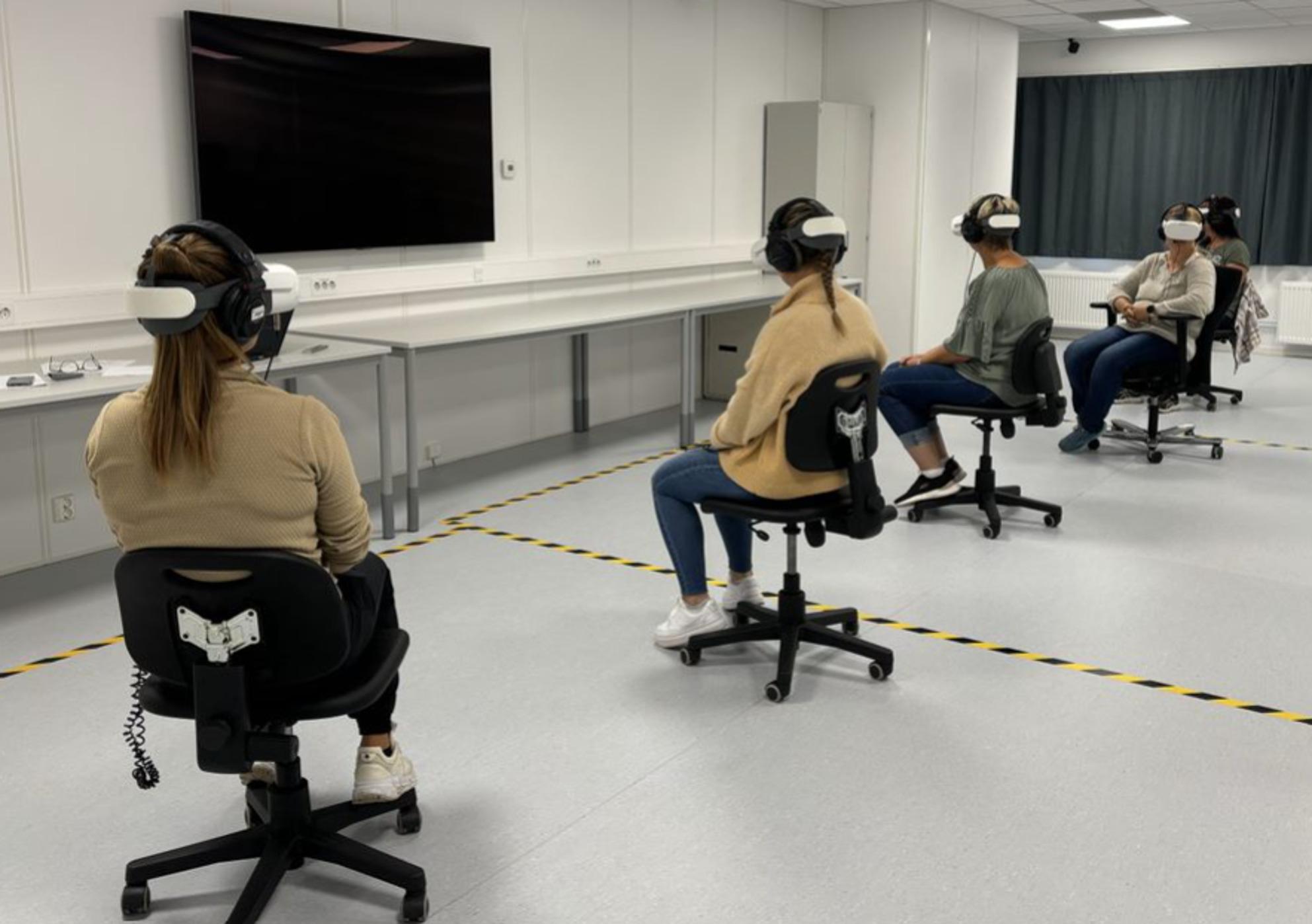
Participants Watching a 360^o^ Video. (Permission has been obtained)

Second, the participants were divided into three groups consisting of four, five and five people, respectively. The learning intervention started with each of the three groups watching one of the three 360° videos (Table 1). Before the participants put on their VR headset, they were given a short introduction to the content of the scenario. While one group watched their assigned video scenario, the other two groups were given a tour at the campus. During the tour they got to mingle with their assigned group. The VR session was facilitated by the co-authors (MHS, KLM), the project manager and a technician. When watching the 360^o^ video, the participants sat on chairs with wheels (Fig. 1). They received assistance in putting on the VR headset and headphones, as the audio equipment was not integrated into the glasses. The technical equipment used was the HTC VIVE Pro Eye. Each participant had the opportunity to watch the 360^o^ video two to three times. Watching the video took approximately 10 min.

Before working through the six steps of the CME model, the participants were encouraged to describe what happened in the 360^o^ video. The intention was to establish a common understanding of what happened in the video scenario. In addition, the last step in the CME model was followed by an application phase, in which the participants were asked how they could put their learning into practice. The description and application phases were facilitated by university staff. The groups of participants were responsible for carrying out the six steps of the structured reflection using the CME model. The purpose of leaving this part for the participants was to facilitate active participation and independent reflection. University staff were present during the group-based reflections to assist if necessary. In line with recommendations from previous research [36], 45 min were set aside for the reflection session after the participants viewed the VR scenario assigned to their group.

The learning intervention was similar for each group, with the only difference being that they watched different 360^o^ videos. After the ethical group reflection session, the participants were served lunch before moving on to focus group interviews (FGIs), which is the data explored in the current study. The FGI’s aimed to explore how the social care staff experienced using the teaching and learning approach (combination of 360° videos and ethical reflection) as a potential tool to enhance promotion of self-determination and preventive dietary practices for people with intellectual disabilities.

## Methods

### Study design

In this case study, we employed an explorative interpretive qualitative design through the testing of a learning intervention. Data was collected through three focus group interviews (FGIs). According to Krueger and Casey [38], FGIs are effective for gathering a diverse array of perspectives and emotions that participants may have regarding a particular topic. Moreover, FGIs can create synergy that exceeds the potential of individual interviews. For example, it allows for dynamic interactions where participants can build on each other’s ideas, clarifying and expanding upon thoughts in real time.

### Participants

The 14 participants in this study worked as professionals in social services in direct care of people with intellectual disabilities. The participants had different health and social education and work backgrounds, but no one had specific leadership or supervision responsibilities. The participants were recruited from the collaborating Norwegian municipality and included two municipality-based supported housing facilities for people with intellectual disabilities. None of the researchers had worked in the two housing facilities or had prior acquaintances with the participants. The participants were recruited by a member of the research team from the municipality in collaboration with the heads of the supported housing facilities. Purposive sampling was used based on the following inclusion criteria: The participants had to be working in a supported housing facility for people with intellectual disabilities. In such supported housing facilities, the professionals’ daily tasks typically include directly assisting people with intellectual disability with managing hygiene, cooking meals, grocery shopping, providing social and relational support, transportation, and organizing recreational activities such as sports participation. In addition, professionals in such services can have additional responsibilities, including managing medications, collaborating with the next of kin, planning health follow-ups, and handling administrative tasks such as finances. Depending on the supported housing facility, the main focus of the professionals is to increase independence, support self-determination, and increase the residents’ quality of life through milieu therapeutic work.

There were no criteria regarding participants’ employment percentage or number of years of experience. The managers of the housing facilities first orally informed their staff about the project. Potential participants also received written information about the study and their right to withdraw at any time, as well as an informed consent form. Before the intervention started, all participants who voluntarily agreed to participate in the study filled in an informed consent form. Fifteen individuals agreed to participate, but one participant was ill on the day the learning intervention was scheduled to take place. The background information of participants is described in Table 3 below.

**Table 3:** Background information of participants

	Total	*n* = 14
Participants		
	Male Female	*n* = 1 *n* = 13
Age		
	25–50 50–65	*n* = 11 *n* = 3
	Mean age Median age	41 years 36 years
Educational level of participants*	Minimum three years university degree Skilled workers (specialized education in high school)	*n* = 8 *n* = 6
Years of work experience	1–15 years 15–31 years	*n* = 10 *n* = 4
	Mean years of experience	*n* = 11 years
	Median years of experience	*n* = 8 years

Note. *Of the eight participants with bachelor’s degrees, seven of them were authorized health personnel (six in social education, one in nursing). One had a degree within another relevant field. The title “skilled worker” is earned through completion of specialized education in high school, followed by an apprenticeship and a practical exam

### Data collection

The data were collected through semi-structured FGIs. The interview guide (Additional File 1) was specifically developed for this study by the research team. Using the interview guide, participants were initially asked to describe their experiences within the VR scenario, including their emotional reactions and reflections. They were also asked whether they could relate to the content of the VR scenario. The questions further explored participants’ experiences with use of VR technology, the structured ethical reflection model and their general experiences of using the teaching and learning approach (using 360° videos in combination with ethical reflection). Additionally, participants were asked about the perceived usefulness of the learning intervention. All of the focus groups were conducted face-to-face at the university campus by the first and last author (HMJ and KLM) and the project manager and were recorded with a separate offline audio recorder. All three interviewers had a member of the project team with them, who could, if necessary, ensure that the interview guide was followed and necessary prompts were provided. Two of the moderators were members of the project team. The recordings lasted from 30 to 50 min, with an average duration of 37 min.

### Analysis

All of the interviews were transcribed verbatim by a professional and reviewed by our project manager. To ensure the confidentiality and anonymity of the participants, possible identifiable details in the data were anonymized, and the participants were assigned numbers. To explore the patterns and complexity in the participants statements, an inductive data analysis was conducted using the six-phase reflexive thematic analysis (TA) developed by Braun and Clarke [39]. This approach acknowledges that data analysis and knowledge generation are inherently subjective and situated, meaning that these processes are influenced by the researcher’s perspectives, experiences, and context. Initially, the researchers became familiar with the dataset (Phase 1), before coding the data within tables in Microsoft word (Phase 2). The coding was revised multiple times and then it was grouped to identify clear patterns and generate initial themes (Phase 3). Three themes were generated by refining and naming initial themes (Phase 4 and 5), before writing the report (Phase 6) [39]. During phases 3 to 5, themes were produced through active collaboration among the researchers, to interpret the data in relation to the research question at both semantic and latent levels.

The researchers (HMJ, MHS, EMIE, MCS and KLM) who participated in the analysis work within nursing- or social care education. The first author (HMJ) is an intensive care nurse and has a PhD in developing digital learning resources for education. MHS is an associate professor and social educator. KLM is an associate professor with an MSc in Psychology. EMIE is a social anthropologist with a PhD in Childhood studies. MCS is a social educator with a PhD related to digital technology design and social inclusion of people with intellectual disability. The first author (HMJ) conducted the initial coding. Thereafter the initial coding was reviewed and discussed with the second and last author (MHS and KLM). This was an iterative process which drew on the diverse perspectives and experiences of the three researchers, to enhance understanding, interpretation, and reflexivity, rather than to achieve consensus about coding. Additionally, all authors reviewed and discussed the themes and subthemes to enhance understanding and to clarify the final results. These insights were utilized in defining the final themes and subthemes. AMF, who is an occupational therapist and works as head of a unit for habilitation within municipality social care services, only took part in this last step. Finally, all authors discussed and commented on the content of the paper (although it was mainly written by HMJ, MHS and KLM). An example of the analysis process is presented in Table 4. The manuscript preparation adhered to the COnsolidated Criteria for REporting Qualitative Research (COREQ) checklist [40].

**Table 4:** Example of the inductive content analysis process

Participant Statement	Code	Theme
I could [using the VR headset] imagine myself in my own role around it and my own decision. Related to it. And then rather hear what others think afterwards (FGI-3).	Perceived advantages	Satisfaction with the teaching and learning approach

### Ethical considerations

This study was carried out in accordance with the ethical principles of the Declaration.

of Helsinki [41] and relevant guidelines and regulations for collection, handling and storing of data. The study was approved by the Norwegian Center for Research Data (nr: 970403), and the faculty’s ethical board (nr: RITM0239863).

An important ethical principle in research is that participation in a study should be voluntarily [41]. To prevent putting any pressure on staff to contribute, they were ensured through oral and written information that it was voluntary to contribute, and that participants could decline to participate or withdraw at any time without any consequences. Another important ethical principle is that participants have the right to confidentiality [41]. Unfortunately, in this study we were dependent on assistance from unit managers to recruit participants. Since the participants was offered the opportunity to participate in the intervention during their working hours or receive hours of credit if they participated on their day off, this made it difficult to keep their participation confidential to their unit manager or the research member from the municipality. Consequently, to ensure the anonymity of the participants, no demographic characteristics about the participants in the three separate FGIs is provided in the presentation of the results and each FGI is treated as one unit of analysis. Thus, when we present direct quotations from the FGI interviews (translated from Norwegian into English) in the results section, they are marked only with the FGI number [FGI-1, FGI-2 and FGI-3].

The member of our research group from the municipality (AMF) have been a coauthor of this paper. To maintain the anonymity of the participants in the analysis of data, this coauthor did not read any transcripts of the data. She contributed only to the discussion and interpretation of the final results and the reading and approval of the final manuscript.

## Results

Based on the analysis of participants’ statements about their experiences with the learning intervention we generated three main themes: (a) the opportunity to experience and immerse oneself in a challenging situation, (b) the opportunity to expand perspectives together, and (c) satisfaction with the teaching and learning approach. Each theme included different but intercorrelated aspects that were sorted into subthemes. Thus, the subcategories are not strictly discrete. The main themes and subthemes are provided in Table 5. The subthemes are further described below, with quotations from the participants in FGI-1 (Lunch), FGI-2 (Shopping List) and FGI-3 (At the Grocery Store).

**Table 5:** Main themes and subthemes

Main Themes	Subthemes
The opportunity to experience and immerse oneself in a challenging situation	• Feeling present in the situation • The VR scenarios evoked emotions and critical thoughts • Recognising oneself in the situation
The opportunity to expand perspectives together	• Increased awareness of how others and oneself act and think • Lack of knowledge as an advantage to reflection
Satisfaction with the teaching and learning approach	• Use of the structured reflection model (CME) • Combining 360° videos with CME • Practical considerations

### The opportunity to experience and immerse oneself in a challenging situation

This theme relates to the unique opportunities that use of 360 ° videos provided in terms of experiencing challenging situations and enhancing emotions and self-awareness. The feeling of closeness to the situations and immersion also evoked feelings of wanting to intervene.

### Feeling present in the situation

Most of the participants shared that they enjoyed the 360° videos. Several participants in the FGIs stated that they felt present in the scenarios, as they were able to change focus and view the whole environment. For example, one participant commented, ‘You felt like you were sitting in the living room there, witnessing the situation, truly’ (FGI-2). Other participants agreed, and some suggested that it might not have been as intense if they had viewed it on a computer screen. For example, several participants in the three FGIs argued that by using the VR glasses, the view became more enclosed and focused. As one participant put it, ‘I felt like I was near them, so that it was somewhat easier to relate to the two people in a way… I felt more connected to the situation and started to immerse myself in it’ (FGI-1). It was also discussed how using a VR headset may prevent distractions from the environment. One participant commented as follows:

I found it very convenient to wear VR headsets because I am very sensitive to distractions. Wearing VR headsets meant I couldn’t see how others around me were moving or reacting to the video. This allowed me to focus intensely on watching the video itself, without any emotional input from those around me (FGI-3).

Only one out of all the participants expressed that it was frustrating not to be able to view from all angles in the scenarios and see both people in the scenario at once. This participant wished to be able to see the reactions of both people at the same time and that the scenarios could have been viewed on a large screen. However, most of the participants in the three FGIs argued that watching the 360° videos with a VR headset could be especially beneficial for new employees, as captured in the following comment: ‘It can be difficult to explain to new employees what dilemmas might be like. So it’s really beneficial to use VR to actually experience and stand in it a bit yourself’ (FGI-2).

### The VR scenarios evoked emotions and critical thoughts

The participants in all three FGIs shared different emotions that they experienced while watching the VR scenario. In the ‘Lunch’ group, the participants mentioned being provoked and feeling sceptical and critical about how the staff member acted. In this scenario, the resident was not involved in making his own lunch. One participant said, ‘What made an impression on me was when she removed the mayonnaise from the slice of bread. I found that degrading; it was sad, disappointing and a bit depressing to watch’ (FGI-1). Furthermore, many of the participants in this FGI expressed that they felt stressed because the staff member did not take enough time.

Participants in the ‘Shopping List’ group expressed that they felt sorry for the resident and hurt on his behalf, as the patient’s wish to have sausages for dinner was not heard. They argued that the staff member acted disrespectfully towards him. Some also shared that they felt frustrated as they thought the staff member completely overruled the resident’s preferences by putting chicken on the list instead. One participant commented, ‘It is easy to override residents. You have a bit more power when you’re a healthcare professional. We saw that there. Even though you shouldn’t really have it’ (FGI-2). Other participants told they felt stressed and powerless because they were not able to intervene. At the same time, some of them felt curious about why she acted the way she did. Was it because she was new and did not know better, or were these her own attitudes and values?

Some participants in the ‘Lunch’ and ‘Shopping List’ groups discussed how the staff member violated the resident’s right to self-determination, as she decided for the resident what he was allowed and not allowed to do. Similar to the other FGIs, participants in the At the ‘Grocery Store’ group had various reactions to the 360° scenario. Some said they felt a bit stressed when the resident and staff member in the scenario approached the shelf with candy and potato chips, as they felt something was going to happen. Two of the participants in this FGI thought the scenario was a bit funny. One of them said, ‘The little trick he came up with, you can go and get the milk, and then I’ll take the potato chips, I do think it’s kind of fun’ (FGI-3). Only one of the participants in this group was surprised by the trick. Some participants also felt a bit sad because the shopping outing did not go as planned, but at the same time, they were happy and relieved that the situation did not escalate. Only one of the participants in all the three FGIs did not feel any particular emotions when viewing the VR scenario.

The feeling of closeness to the situation and the critical thoughts about how the situation was handled evoked feelings of wanting to intervene among several of the participants in all three FGIs. Many participants expressed that they felt an urge to guide the young staff member. For example, participants in the ‘Lunch’ group expressed that the staff member should have taken more time, showed more respect, involved the resident more in decisions and given him more choices. In the ‘Shopping List’ group, one of the participants said, ‘There was an urge to tell the person how to do it. If I had had an apprentice with me, or someone in training, and saw something like that happening, I would have stepped in and stopped it. That urge definitely arose’ (FGI-2).

Participants in the ‘At the Grocery Store’ group shared that they thought of alternative ways of handling the situation in the grocery store and wished they could have guided the staff member. One thought, ‘Oh no, do it like that instead, it’s okay’ (FGI-3). They also mentioned that staff work according to ‘certain guidelines’, while at the same time, one must come to an agreement with the resident and potentially find a middle ground within certain limits: ‘You know when you work often with them, you have an agreement, or how can I say, you find a way of working with them. So, you know their limits, and you know your own limits’ (FGI-3).

### Recognising oneself in the situation

The participants in the FGIs expressed that it was easier to recognise themselves in the situation by viewing the 360° video than just by reading a case on a piece of paper. Several of the participants in all three FGIs expressed that they had been in similar situations and shared some thoughts. According to one of the participants in the ‘Lunch’ group, ‘these kinds of situations are recognisable and happen to us daily’ (FGI-1). Similarly, participants in the ‘Shopping List’ group shared that such situations happened at least once per week – that is, situations where the resident wants something that is not health promoting. One of them argued, ‘But it’s really about providing guidance [from staff]’ (FGI-2). This was echoed in the following comment from another participant: ‘We make a lot of agreements and refer to these agreements to ensure things are carried out. And of course, if they don’t want to, we try to motivate, explain and guide them in that direction’ (FGI-2).

Several participants in the ‘At the Grocery Store’ group said they recognised the situation and the feeling of being in the staff member’s situation. One said, ‘I could somewhat relate to her, that one can become a bit stressed in such a situation. I thought, is this going to escalate, will there be an outburst?’ (FGI-3). They all expressed that they felt empathy for the staff member in the scenario. Another participant commented, ‘It is incredibly painful to be in that situation when things go wrong’ (FGI-3). Similarly, one of the less experienced participants recognised the feeling of being insecure and shared that she often had to seek guidance from more experienced staff.

### The opportunity to expand perspectives together

This theme relates to how both the 360° videos and using the CME model for subsequent group reflections made participants more aware of own and other participants’ thoughts and actions. Furthermore, the fact that they had little information about the people in the videos promoted more reflections and awareness of the importance of gathering more information before making decisions involving the service users.

### Increased awareness of how others and oneself act and think

The participants in the focus groups expressed a range of opinions about the staff member’s behavior shown in the 360° videos. One of the participants in the ‘Lunch’ group shared the feeling that the staff member imposed too many directives, going beyond mere facilitation: ‘I think the power dynamic was wrong there, that she had a bit too much control and direction’ (FGI-1). Another participant supported this and expressed that the situation was recognisable: ‘I have seen myself fall into the same trap, and I have seen others fall into that trap. It happens very quickly’ (FGI-1). Participants in the ‘Lunch’ group discussed and suggested different explanations for the staff member’s behaviour, such as that she seemed quite young and inexperienced. Some argued that the staff member’s actions could also possibly be explained by her feeling obligated to follow the local regulations, even if this meant acting against her own values. On the other hand, the participants also shared that occasionally residents do not want to follow the plan that they themselves have been involved in creating and have agreed upon and that this was challenging.

The rushed situation depicted in the ‘Lunch’ video made one of the participants think about and share own experiences about how spending enough time with residents matters. This participant commented as follows:

When you take your time, have a good conversation without rushing anything, it suddenly becomes a lot more pleasant. And just sitting down when having a meal – if you have that time, use it! You actually get a lot of useful information. (FGI-1)

It was also discussed in the FGI groups that it is unfortunate and confusing if employees express different opinions to the residents on what is acceptable when it comes to nutrition and food. One participant commented:

Food may be one of the biggest challenges one faces. And because everyone [staff] has such different ideas about how things should be, what is acceptable and what is an appropriate amount versus too much, it’s no wonder that they [the residents] don’t know what to relate to when we can’t even agree among ourselves. (FGI-1)

It was also emphasized by participants within the three FGIs the importance of having experience working with people with cognitive disabilities. One said, ‘When you’ve worked for a few years, you see that you would have done it differently yourself’ (FGI-2). Another participant suggested that staffs’ perspectives are often connected to historical understandings of people with intellectual disabilities: ‘If we had gone back a few decades, or perhaps not even that long, we might have viewed the same ethical dilemma completely differently’ (FGI-1). It was also discussed among the participants how handling situations could differ, depending on the time and resources they have available that day. For example, it was suggested that if they had little time one day, it could be more effective to fix breakfast for the resident instead of making him do everything by himself. However, then it was important to explain to the resident why things were done differently and promise to make it up to him next time.

The participants expressed during the FGIs that the structure of the CME model (Table 2) supported the groups’ initial reflection, first by helping them identify and agree upon the factual elements of the 360° video scenario they had watched. For example, in the first step of the CME model, the participants agreed that the 360° videos demonstrated ethical dilemmas concerning balancing the residents’ right to self-determination with the duty of staff to provide sound professional services. Both the less and more experienced participants expressed that using the CME model for group-based reflection had made them aware of perspectives that they had not previously thought about and that this had expanded their views. For example, some participants expressed that using the structured CME model made them aware of the perspectives of other parties involved (i.e. relatives, general practitioners, etc.,), which perhaps had not been considered at all. One of the participants in the ‘Shopping List’ group shared that the ethical reflection had changed the participant’s opinion about the staff member in the scenario, commenting as follows: ‘I had a negative attitude towards the staff member right up until we discussed the situation in the ethical reflection session. Then I managed to turn it around and see her side as well’ (FGI-2). In addition, the group-based ethical reflections also made the participants aware that there are many so-called grey zones or situations that lack clear guidelines or boundaries, for example, using coersion versus being actively insisting. Participants argued that in such dilemmas, it is important to use ethical reflection.

By using the CME model, participants also shared that they became more aware of own actions and various alternative actions. One participant argued that it can be difficult to break out of one’s own patterns of behaviour: ‘You can easily continue in the same track. It is difficult to break free regardless of age, really. It’s tough as well’ (FGI-1). Another participant in the same group concluded; ‘The use of the CME model brings out a more holistic view of the scenarios and sheds light on things one usually doesn’t think about’ (FGI-1).

### Lack of information as an advantage in promoting reflection

During the FGIs, all participants acknowledged that by watching a 360° video versus experiencing the situation in their own workplace, they did not know much about the background of the situation. For example, the participants in all three groups noted that it could have been an advantage to have background information about the staff member in the scenarios, such as her training (or lack thereof), to get insight into why she handled the situation as she did. In addition, participants lacked information about the resident and any possible health issues this person might have. Considering the lack of this information, one participant commented: ‘One gets insecure about how it could have been handled better and given the resident more self-determination and choice’ (FGI-1). However, after using the CME model for group-based ethical reflection, several participants in the FGIs pointed out that the lack of information actually had been an advantage. One participant suggested that the lack of information had made the reflection even better: ‘It made us reflect on many scenarios […] So it was kind of helpful not to know, too’ (FGI-1).

Based on the group-based ethical discussions, most participants argued that in daily practice, background information and information related to the individual resident concerning nutrition are essential to obtain to be able to make judgements and decisions related to self-determination and nutrition. Following up on this argument, one participant pointed out the importance of knowing the individual resident well: ‘I think that a lot is about how well you know the resident, including their history, what they can do, and what they like. It’s about being genuinely interested in who they are’ (FGI-1).

### Satisfaction with the teaching and learning approach

This theme relates to participants’ perceptions of the teaching and learning approach, its potential as a learning tool, perceived advantages and disadvantages, as well as participants’ recommendations regarding its organization.

### Use of the structured reflection model (CME)

Most of the participants in the three FGIs expressed that they had experienced advantages with using the CME model. For example, it was an advantage to use it to structure the discussion, because they had to consider all the factors (elements in the CME model) step by step before they could decide on alternative actions in the scenarios.

It was also discussed in the three FGIs how using the structured reflection model could potentially help reveal any negative culture and possibly disrupt inappropriate behaviour patterns amongst staff at their respective workplace. A participant who had worked for a long time in the service stated that experienced staff might develop quite fixed behaviour patterns, and that it was beneficial to be challenged in this regard: ‘If a person has entirely different opinions from the others, they must actually justify this based on the model’ (FGI-1). As part of this discussion one of the young participants proposed that use of the CME model also was useful in terms of focusing on current legislation and recommended practices in the social services. The participant argued that new graduates and newly employed individuals may have different perspectives but often choose to be considerate and loyal to the system.

Only one participant in FGI-2 and one in FGI-3 (the ‘Shopping list’ and ‘At the Grocery Store’ groups) expressed that they were not particularly fond of using the CME model in their workplace. The participant from FGI-2 said: ‘We used it before, but then we stopped using it because we felt it became too framed in a way. We benefit more from just discussing the topic, instead of categorizing it with laws and such’. Among other disadvantages that were discussed in the three FGIs was the time it takes to go through all the steps. An additional disadvantage that was pointed out in two of the FGI groups was that it could be complicated to use: ‘There are so many points you have to understand in order to be able to discuss it’ (FGI-3). For example, not everyone knew of or remembered the current laws and regulations related to residents’ rights or different terms or ethical perspectives, which required some people to use Google. Consequently, it was suggested that it was beneficial that some in the group discussions could explain the ethical issues to the others.

In addition to the mentioned disadvantages, participants in all three FGIs argued that it can be particularly challenging to go through all the steps in the CME model before reaching the discussion of possible solutions. In relation to this discussion, one participant suggested using the CME model in a slightly different order; ‘Maybe one can talk loosely and casually about it first, and then one can sort of fit it [the discussion] into the model afterwards, and then apply the theory very carefully. Because then people know a little more and have thought about it first’ (FGI-3).

### Combining the 360° videos with the CME model

Even though participants pointed to some disadvantages of using the CME model, most participants expressed that they liked using the 360° video in combination with the structured ethical reflection and found it useful. For example, some participants commented that since the staff might have slightly different values and reactions to the situation in the 360° video, it was useful to be able to share and describe their own reactions. Similarly, participants discussed how the videos and the ethical reflection made them become aware of their own values and care. One said, ‘I think you become more aware of how you provide health care’ (FGI-2). Following these arguments, it was suggested that staff members who work together as a team would benefit from viewing the scenarios together and having these discussions. Similarly, many participants suggested that going through such a learning programme would be extremely useful for recent graduates with little experience. This was confirmed by a participant who was a newly hired employee. Others also recommended that the teaching and learning approach should be a mandatory part of the educational programme for social care personnel to prepare them for clinical placement and future work. At the same time, several participants in the three FGIs argued that it had been both fun and useful for the more experienced ones to use the 360° video for learning, but underlined that viewing 360° videos should be followed by an ethical discussion.

The participants in all three FGIs also found the three VR scenarios and the ethical discussions related to self-determination and nutritional health relevant. One participant commented as follows:

I think this is very relevant, and I feel that food and nutrition are among the most challenging ethical dilemmas we face on a daily basis, really […] so it’s really nice to have discussions around it and [get] different input from others that one doesn’t encounter daily. (FGI-3)

### Practical considerations

In relation to the organization of the learning intervention, some participants in the three FGIs expressed that they wished they had received more information about the approach and purpose beforehand. However, regarding viewing the VR scenarios, the participants thought it was an advantage to have watched it twice before the discussion. They also liked that they had an opportunity to mingle with the other participants and get to know them before the reflection session started because this made it easier to discuss the ethical dilemmas and alternative ways of handling the situation in the scenario. Furthermore, the participants appreciated receiving a piece of paper with keywords related to the CME model, which they felt made it easier to structure the discussion.

The participants’ opinions about including staff from two different organisations in the three intervention groups varied among the participants. Some expressed that it was a bit quiet in the group reflection at the beginning, since they were used to discussing ethical dilemmas only in staff meetings with people they knew. However, most participants felt that the mixed groups were an advantage:

I thought it was really nice to have the ethical reflection with personnel you don’t know, with people working at another place. You may be presented different viewpoints that you have not considered before. (FGI-1)

During this discussion, several participants also shared that they appreciated that they were organised in small groups. One participant commented:

When there are fewer people in a group, speaking up doesn’t feel as intimidating. If we were all together, someone might not say anything. Being in smaller groups can also make you feel like you’re participating more. (FGI-2)

The participants in all three FGIs also expressed different opinions about travelling to the university campus to attend the teaching and learning intervention. Some expressed that it might be more effective to be able to view the 360° videos and participate in the discussion on ethics at their workplace. Then it could be done during their shift and as part of a regular staff meeting, and perhaps more staff members would have an opportunity to view the videos. It would also reduce costs related to sending staff to the university. Others preferred coming to the university campus because they could focus completely on viewing the VR scenarios and participate in the structured ethical discussion without any interruption.

## Discussion

In this study we explore how social care staff experience the use of 360° videos combined with ethical reflection as a tool to enhance the promotion of self-determination and preventive dietary practices for people with intellectual disabilities.

Participants’ reported a strong sense of immersion and presence when watching the 360° video, consistent with previous research [21, 22, 30]. Although 360° videos viewed with VR headset has been shown to be more immersive than watching 2D videos on a monitor [42], one participant preferred a standard flat screen. This suggests that 360° video formats may not be universally preferred and can be experienced as distracting by some viewers [21, 23]. Still, our results showed that watching the 360° videos evoked several emotions among the participants, consistent with previous research [25, 28, 43, 44]. The participants’ emotions mostly had a negative valence and were accompanied by critical statements about how the actor handled the situation. For example, in the ‘Lunch’ and ‘Shopping List’ scenarios, the participants felt that the staff member exerted influence on the resident against his expressed will, in accordance with ‘informal coercion’ [19]. In addition, many participants argued that the staff member in the ‘At the Grocery Store’ scenario did not offer enough guidance or use enough inviting practices [19]. Consequently, the staff member’s actions and attitude provoked uncomfortable emotions and responses from the participants, which can be interpreted as another expression of perceived relevance and the immersive qualities of the 360° video [22, 25, 28]. Most importantly, these responses underscore that the 360° VR scenarios managed to evoke ethical sensitivity, reflection and ethical judgement related to the facilitation of self-determination of people with intellectual disabilities, aligning with prior findings on reflective learning through 360° videos [21, 29, 44]. However, based on our data, we cannot tell whether this in-situ increase in ethical sensitivity will carry over to real practical situations later, so further research is warranted. On the other hand, the increase in ethical sensitivity might simply reflect that participants were asked to think about ethics, which generated an expectation to be critical of what happened in the VR scenario. To avoid this, the initial instructions could be to ask participants to reflect broadly (e.g., on their impressions or choices) without mentioning ethics or dilemmas.

The 360° videos combined with using the CME model, promoted additional self-awareness and internal reflection [35, 44]. An example of participants’ self-reflection – which may be viewed as a paradox – was that even though the participants experienced negative emotions and had critical thoughts about the staff member’s use of informal coercion in the situation portrayed in the scenario, they also stated they could recognise themselves in the situations: ‘I have seen myself fall into the same trap’. This self-awareness and self-reflection is in line with the concepts of ‘reflection in and on action’ [35] and Kolb’s experiential learning theory [34]. Interestingly, the participants did not suggest similar or different forms of informal coercion as a possible solution to tackling difficulties with promoting a healthy diet. Still, while it should be underlined that the participants were predominantly occupied with reflecting on the theme of facilitating self-determination and the ethics of using coercion with people with intellectual disabilities, they hardly discussed how staff are on the other hand bound by law to provide necessary care to prevent ‘significant harm’ (i.e. assist residents in avoiding nutrition related health issues). In sum, these results indicate that the participants showed openness to reflect on their own practice, but also in turn revealing possible limitations in knowledge of relevant legislation and the potential of using informal coercion as a milieu therapeutic approach. To increase promotion of self-determination and preventative dietary practices in line with sound professional practice, additional measures may be needed (i.e. to increase knowledge of legal conditions). Nonetheless, our results support evidence of the importance of practice-relevant reflection [20, 28, 36]. This also aligns with Kolb’s [34] proposition that experiential learning depends on both the quality of the experience and meaningful reflection [34, 45].

Some of the participants stated that the CME model was ‘too comprehensive’ for practical use and that they had implemented more ‘informal’ ethical discussions at their workplaces. This result suggests that staff may avoid using the CME model because of limited knowledge of legislation and ethics, or that ethical reflection might not be a primary focus in their daily work. Such issues are reflected in a report from the Norwegian Board of Health Supervision [46] which identified serious service failures with potential consequences for users’ health and quality of life. Key findings included inadequately tailored personal assistance, insufficient staff competence and training, unsystematic service provision, poor information flow, and limited user involvement, with relatives and guardians insufficiently informed. These shortcomings also applied to dietary support. On the other hand, the services where our participants work are generally clinical environments characterised by high pace and time constraints. It is therefore not surprising that some may prefer less structured and rapid reflections and consequently find the CME model too comprehensive. However, the structure offered by the CME model, or a similar ethical reflection model, is more likely to ensure relevant considerations and also, as discussed above, reveal gaps in critical knowledge.

The result that participants’ expressed a desire to intervene in the 360° scenarios suggests moral discomfort alongside confidence in their own competence, indicating relevant professional knowledge and motivation [44]. However, this also highlights challenges in translating knowledge about nutrition, self-determination, and legislation into clinical practice [4, 5]. This result supports calls to prioritize increased knowledge on the balance between the protection of rights and the nutritional health for people with intellectual disabilities [5, 6, 47]. Thus, more research is needed to clarify and guide staff in practical assistance in health promotion to people with intellectual disabilities. There is especially a need for more research on the grey area between total self-determination and possible ignorance on one hand, and paternalism and coercion on the other.

Our study showed that both ‘Reflection in and on action’ [35] was enhanced through the ethical group-based reflections. Following the CME model [36], the discussions helped the participants identify relevant stakeholders and their perspectives as well as the ethical challenges and principles involved. Use of the reflection model prompted them to discuss norms, legislation and different options for action and helped them identify ineffective and disrespectful staff strategies. In regard to legislation, some participants actively sought additional knowledge by using Google, a positive outcome given the knowledge gaps in this field [3]. However, patient and user rights legislation was largely absent from discussions, suggesting that activating relevant legal knowledge in combination with ethical knowledge (i.e., ethical theory) prior to viewing the 360° videos may strengthen learning outcomes. A similar approach as has been proposed in other studies where participants have lacked relevant knowledge prior to a learning intervention [28, 36].

Participants’ knowledge was developed not only through individual reflections [34] but also through the collaborating group-based reflections [23], which helped them consider new perspectives and rethink their own practices, as confirmed by other studies. As one of the participants commented: ‘…I managed to turn it around and see her side as well’. This shows that structured group-based ethical discussions have the potential to inspire some participants to rethink their own practices and attitudes [20, 36]. One participant also suggested that staffs’ perspectives could be connected to historical understandings of people with intellectual disabilities. Different historical perspectives may be related to the shift from a deficits approach to intellectual and developmental disabilities to a more strength-based approach that focus more on quality of life and self-determination [13]. Therefore, a group discussion with colleagues can be beneficial, because when staff share their viewpoints openly, they gain new insights [23, 44]. However, in our study, the groups were heterogeneously composed (from different service units), but the participants still valued the diversity of perspectives it provided. We therefore believe that increased reflective practice may support changes in behaviour and professional understanding.

Many participants reported needing more contextual knowledge about the resident, the staff member and the situation portrayed in the 360° videos to make sound judgements. Lack of information can be taken as a criticism of the short 360° videos themselves or lack of supplementary information. It may also be an expression of participant wanting to avoid making difficult decisions possibly compromising users’ self-determination. However, it can further be understood as demonstrating a general point about working with people with intellectual disabilities. When a problematic situation arises, a general recommendation is to take sufficient time to assess the situation before taking action and to base any intervention on sound knowledge of the situation [3–5]. The participants’ need for more information can thus be interpreted as supporting this general recommendation.

Participants reported that combining the 360° videos with structured group reflection was both fruitful and essential, consistent with other research [28–31, 33]. In line with Karlsen et al. [36], the participants also proposed that applying the CME model at the service level could foster an organisational culture of ethical reflection and shared understanding of best practices concerning food, nutrition and self-determination for people with intellectual disabilities. For example, in the CME-based structured reflections, the participants revealed rich perspectives and competence in supporting residents through their suggestions for intervening in the scenarios and for offering guidance to the staff members portrayed in the 360° videos. This may be a potential source of knowledge for reducing unethical use of power and coercion. However, to unlock this potential, this responsibility must be addressed at both the policy and organisational levels so that the responsibility for making challenging decisions is not left to individual staff members [19, 47].

Despite the positive feedback on the teaching and learning approach, we acknowledge that the learning and teaching approach has some limitations. The 360° videos supported observation and reflection [21], but did not allow interactivity, such as being able to intervene in the situations. This passive observation role and lack of interactivity is considered one of the most important limitations of 360° videos [21, 22]. As one participant preferred not using the VR headset, combining 360^o^ and traditional 2D videos may be useful in teaching contexts, as suggested by Kulke and Pasqualette [42].

In relation to organization of the learning intervention, the participants expressed different views about the composition of the groups (mixed or homogeneous) and group size, with some favoring mixed groups for diversity, and others preferring homogeneous groups comprised of staff from the same workplace. To ensure diversity of perspectives in the groups, but at the same time prevent discomfort among the participants, we created small mixed groups and ensured that the group members got to know each other before the group reflection started. This is also in line with the recommendations offered in other research [20, 33, 48].

Many participants expressed a desire to implement this approach in their own workplace. There may however be some challenges regarding the scalability of such a learning interventions within participants’ own workplaces. These include cost of VR devices, potential technical issues, and limitations in general technical and professional facilitation prior, during and after the intervention. An additional and important trade-off experienced by the participants in using the CME model for guiding reflection was that it was time-consuming. Time constraints are identified as an organisational factor that may be a barrier to successful reflection [36]. Nonetheless, finding ways to elicit and manage professional creativity through group reflections among staff members can be a way to further develop professional identity [44] and encourage staff to strive to maintain professional ethical standards [36]. However, this relies on opportunities to express and define problems and explore solutions together in order to establish routines for food, meals and nutrition together with the residents [3–5].

### Methodological considerations

In this qualitative study, we have ensured values of trustworthiness, including credibility, dependability and transferability [49]. The criteria for credibility may be understood as the ways in which our research accurately reflects the participants’ perspectives and experiences. By choosing FGIs, our intention was to capture the range of different perspectives or feelings that the participants had about the teaching and learning approach. To ensure a range of perspectives, we recruited participants with diverse health- and social education- and work backgrounds. Furthermore, to avoid participants feeling obliged to provide only positive evaluations of the intervention and its usefulness, we informed them that we welcomed all kinds of feedback, including criticisms and suggestions for improvement. In addition, to prevent confirmation bias we ensured that the intervention facilitators conducted interviews with a different group during the group interviews. Despite these precautions, few negative remarks about the intervention were made by participants. Thus, the uniformly positive results may indicate possible methodological bias. For example, the selection of participants by the municipality may have resulted in an overrepresentation of staff who are technologically positive and particularly reflective. Furthermore, the fact that the focus groups were conducted immediately after the intervention may lead to bias such as recency (participants overemphasize the importance of recent experiences) and social desirability (participants strive to appear reflective and look good to other participants and the researchers).

The use of FGIs may also have some limitations [38]. For example, some participants may not comment on all the questions, while others may comment several times on one issue. Being aware of this, the researchers did rounds of questioning to ensure that all participants contributed their thoughts and perspectives. In addition, we chose small groups of four to five participants to ensure that the participants felt comfortable speaking up. In the results section, we included quotations from all three FGIs as well as quotations that show the participants’ different perspectives. Since the purpose of FGIs is to capture the range of different perspectives or feelings that participants have about a topic, rather than providing frequencies by numbers or percentages [38], the participants’ responses are presented in the manuscript using descriptors such as ‘some participants’, ‘many participants’, ‘most participants’ and other adjectives.

Dependability was achieved by using the same interview guide in all the FGIs. The interview guide for the FGIs was developed by the authors, who together have extensive expertise in social care education and social work, research related to people with intellectual disabilities, qualitative research and digital learning tools.

In relation to the credibility of the data, the three authors HMJ, MHS and KLM conducted the initial data analysis. To prevent possible bias from researchers’ preunderstanding and assumptions (those with background from social services and social education), a researcher from nursing science (HMJ) who was not familiar with social care services did the initial coding and produced initial themes. The results of the analysis were also discussed with the interdisciplinary coauthors until agreement was reached. Furthermore, as being the developers of the 360^o^ videos and intervention, the authors HMJ, EMIE and MCS) were especially careful to avoid their involvement biasing the analysis or reporting of the results. Accordingly, the authors endeavoured to report both the strengths and limitations of the intervention in the manuscript. Thus, we argue that being an interdisciplinary team of researchers may be considered a strength of this study.

In relation to transferability, we have described the context, the intervention, the participants and the research process thoroughly. Even if the sample size was small, the data collected from the three FGIs and the 14 participants provided substantial insights relevant to our study. Thus, we argue that information power was achieved [50]. Taking precautions about contextual differences, our results may provide valuable information for planning and executing comparable learning interventions in similar areas of professional practice. Our results are also supported by other research and theory. Therefore, we argue that our qualitative study meets the criteria for trustworthiness.

Concerning strengths of our learning intervention, the collaborative participation of residents, relatives and staff in the development of the videos most likely contributed to participants’ perceptions that the 360° videos were realistic and recognisable. Such a collaborative development is in line with suggestions from Lie et al. [48].

Our study has some additional limitations. The group of participants was not homogenous, so different educational backgrounds and years of experience may have affected their perspectives. However, in this study we chose to consider different levels of experience and perspectives as an advantage to ethical reflections. Due to confidentiality reasons we had to minimize presenting demographics of the participants. However, this has also limited our ability to compare statements and reflections among participants in regard to demographics such as age, educational level and years of work experience. Lastly, the fact that the participants worked as professionals in supported housing facilities in direct contact with people with intellectual disabilities may be considered a limitation or a strength to this study. For example, some might argue that the participants’ earlier experiences and daily work may have biased their view(s) of what occurred in the VR video and, consequently, the structured group-based ethical reflection. However, in contrast, we argue that the participants’ earlier experiences supported more detailed reflections based on real-world events, experience sharing, and promoted dual perspective taking - meaning the ability to both identify with the person receiving care and effectively evaluate their own professional position.

## Conclusions

This study addressed the following research question: How do social care staff experience the use of 360° videos combined with ethical reflection as a tool to enhance the promotion of self-determination and preventive dietary practices for people with intellectual disabilities? Our results showed that the participants in all three FGIs found the content of the VR scenarios and the ethical discussions both relevant and useful. Our results showed that the teaching and learning approach heightened self-awareness, ethical sensitivity, reflection and ethical judgement related to the facilitation of self-determination for people with intellectual disabilities. It was suggested that staff members who work together as a team would benefit from viewing the scenarios together and having these ethical discussions. It could potentially also help reveal knowledge needs, any negative culture and possibly disrupt inappropriate behaviour patterns in relation to promotion of self-determination and preventive dietary practices for people with intellectual disabilities.

Based on the results, the teaching and learning approach seems to be suitable for addressing one of the most challenging ethical dilemmas described by the participants – balancing how much to insist on making healthy dietary choices with a recognition of residents’ right to self-determination. The approach may benefit from adding teaching and/or implementation strategies. What remains to be seen is if and how the learning approach actually contribute to changes in professional practice. Future research should focus on testing the learning intervention in the staff members’ own environment and measuring its effectiveness in terms of increasing their preparedness and confidence, as well as measuring the impacts on the care that staff members provide for people with intellectual disabilities. Thus, the implications for practice is that healthcare institutions should offer their staff regular opportunities to use such teaching and learning approach at their sites or at the university.

## Supplementary Information

Below is the link to the electronic supplementary material.

Supplementary Material 1

## Data Availability

The datasets used/analysed (in Norwegian) in the current study are available from the corresponding author upon reasonable request.

## Abbreviations

CME: Centre for Medical Ethics
CRPD: The United Nations Convention on the Rights of Persons with Disabilities
VR: Virtual reality
3D: 3-dimensional
2D: 2-dimensional
