# Supplementary material for: Training social care staff in promoting self-determination and nutritional health for people with intellectual disabilities using 360o virtual reality videos and ethical reflection: a qualitative study
Source: BMC Health Serv Res. 2026 Mar 16;26:385. doi: 10.1186/s12913-026-14308-5 (PMC13011561; doi:10.1186/s12913-026-14308-5)
Supplement: Supplementary file 1 — Supplementary Material 1 [file 12913_2026_14308_MOESM1_ESM.docx]

**Interviewguide**

- Could you please describe the scenario that you saw in the VR headset?
- How did the situations or conversations make you react and feel? Why?
- Were there any previous experiences that you thought about during or after watching the scenario?
- Could you describe your willingness to help people in the scenario?
- How did you experience using 360^o^ videos and VR headset?
- How did you experience using the structured ethical reflection model (CME)?
- How did you experience the teaching- and learning approach (the combination og 360^o^ videos and ethical reflection) and the facilitation of it?
- How would you describe what you learned today?
- Anything you would like to add?
